# Inter-Regional Proteomic Profiling of the Human Brain Using an Optimized Protein Extraction Method from Formalin-Fixed Tissue to Identify Signaling Pathways

**DOI:** 10.3390/ijms24054283

**Published:** 2023-02-21

**Authors:** Jennilee M. Davidson, Stephanie L. Rayner, Sidong Liu, Flora Cheng, Antonio Di Ieva, Roger S. Chung, Albert Lee

**Affiliations:** 1 Centre for Motor Neuron Disease Research, Macquarie Medical School, Faculty of Medicine, Health and Human Sciences, Macquarie University, Level 1, 75 Talavera Road, Sydney, NSW 2109, Australia; 2 Centre for Health Informatics, Faculty of Medicine, Health and Human Sciences, Macquarie University, 75 Talavera Road, Sydney, NSW 2109, Australia; 3 Computational NeuroSurgery (CNS) Lab, Macquarie Medical School, Faculty of Medicine, Health and Human Sciences, Macquarie University, Level 1, 75 Talavera Road, Sydney, NSW 2109, Australia

**Keywords:** human brain, tissue, neuroanatomic region, formalin-fixed, proteomics, method, signaling, protein, pathways, mass spectrometry

## Abstract

Proteomics offers vast potential for studying the molecular regulation of the human brain. Formalin fixation is a common method for preserving human tissue; however, it presents challenges for proteomic analysis. In this study, we compared the efficiency of two different protein-extraction buffers on three post-mortem, formalin-fixed human brains. Equal amounts of extracted proteins were subjected to in-gel tryptic digestion and LC-MS/MS. Protein, peptide sequence, and peptide group identifications; protein abundance; and gene ontology pathways were analyzed. Protein extraction was superior using lysis buffer containing tris(hydroxymethyl)aminomethane hydrochloride, sodium dodecyl sulfate, sodium deoxycholate, and Triton X-100 (TrisHCl, SDS, SDC, Triton X-100), which was then used for inter-regional analysis. Pre-frontal, motor, temporal, and occipital cortex tissues were analyzed by label free quantification (LFQ) proteomics, Ingenuity Pathway Analysis and PANTHERdb. Inter-regional analysis revealed differential enrichment of proteins. We found similarly activated cellular signaling pathways in different brain regions, suggesting commonalities in the molecular regulation of neuroanatomically-linked brain functions. Overall, we developed an optimized, robust, and efficient method for protein extraction from formalin-fixed human brain tissue for in-depth LFQ proteomics. We also demonstrate herein that this method is suitable for rapid and routine analysis to uncover molecular signaling pathways in the human brain.

## 1. Introduction

The human brain is a highly complex organ with various functions, including higher cognitive functions (e.g., thought, memory, and emotion) that control speech, motor skills, vision, and other processes that are fundamental to life. The human brain’s surface is historically divided into four main lobes, i.e., the frontal, temporal, parietal, and occipital lobes, although the modern atlas includes a much more sophisticated structural and functional parcellation map [1]. The historical (but reductionistic) view is that each of these lobes is mainly related to some specific functions. It is important to note that different areas of the same lobe are specialized for different functions, and that many functions are distributed among several brain regions. Recent research into the brain’s connectome has revealed higher complexity of regulation than the simplified historical functional areas [2].

Omics studies have increased our understanding of the human brain’s functions [3,4]. Genetic variants have been reported to influence the total surface area and thickness of the brain. There are specific genetic influences on inter-regional cortical areas [5]. In a similar fashion to how the genetic architecture influences the human cortex and shapes our understanding of human brain functions, proteomics approaches are increasingly being applied to understand the complex networks of proteins that are co-regulated within the brain. A preliminary investigation of Broadmann areas in one hemisphere of one human brain revealed proteomic similarity within common functional areas (i.e., speech). This suggests that the proteomic map reflects the functional parcellations of the human cerebral cortex [3]. Hence, proteomics offers vast potential for studying the underlying proteome and signaling pathways in different areas of the brain [3,4,6,7]. Proteomic and bioinformatic analyses can be used to identify differentially regulated proteins in different brain regions, which can help elucidate the molecular bases underlying brain functions. Understanding molecular mechanisms in different brain regions can provide insights into brain function in both healthy and diseased states. 

Several proteomic techniques can be used to investigate the human brain’s proteome, including multiplexed quantitative method tandem mass tag (TMT) isobaric labelling [8,9], two-dimensional liquid chromatography coupled with tandem mass spectrometry (2DLC-MS/MS) combined with isobaric tags for relative and absolute quantitation (iTRAQ) [3], matrix-assisted laser desorption/ionization imaging mass spectrometry (MALDI-IMS) [10], parallel reaction monitoring (PRM) [6,11] and liquid chromatography–tandem mass spectrometry (LC-MS/MS) [4,6,12]. Due to the inherent technical limitations incurred while obtaining and storing human brain tissue samples, the appropriate proteomic method must be selected. Samples may be affected by the storage conditions and the age of the sample. Unfortunately, most of these approaches can be expensive and are highly dependent upon quality of tissue, hence the preferred use of fresh frozen tissue. However, with clinical samples, formalin fixation is a common method for preserving human tissue. It crosslinks the proteins in the tissue for long term storage. LC-MS/MS proteomics has been carried out with formalin-fixed, paraffin-embedded (FFPE) prostate, bile duct, colorectal, renal and other human tissues [13,14,15,16]. LC-MS/MS also offers a relatively cost-effective, unbiased label-free quantitative method to investigate the global proteome. Moreover, FFPE archival tissue has proven its utility and comparability to fresh frozen tissue for LC-MS/MS analysis [17].

**Figure 1:**
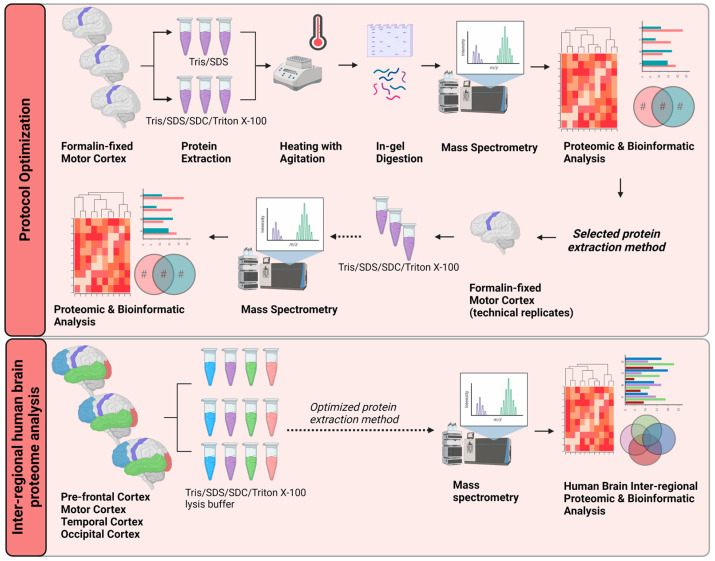
Schematic workflow of the proposed optimized protein extraction from formalin-fixed, post-mortem, human brain tissue for proteomic and bioinformatic analyses. Three separate biological replicates of motor cortex tissue were subjected to two different protein-extraction buffers. Trypsin was used to digest proteins in-gel for liquid chromatography–tandem mass spectrometry. The proteomes obtained from each method were compared by proteomic and bioinformatic analyses. Lysing the formalin-fixed tissue in protein-extraction buffer containing Tris/SDS/SDC/Triton X-100 gave the most robust results. This optimized method was applied to tissue from four neuroanatomical regions (pre-frontal cortex, motor cortex, temporal cortex, and occipital cortex) of three separate post-mortem brains. Proteomic and bioinformatic analyses were carried out to identify functionally linked or neuroanatomically linked cellular signaling pathways. Tris, Tris (hydroxymethyl) aminomethane hydrochloride; SDS, sodium dodecyl sulfate; SDC, sodium deoxycholate. Created with BioRender.com.

In this study, we developed an efficient and robust protein extraction method for LC-MS/MS analysis of formalin-fixed human brain cortex tissue. We present an optimized method to efficiently extract proteins using lysis buffer containing a high concentration of tris (hydroxymethyl) aminomethane hydrochloride (TrisHCl, denoted as Tris), sodium dodecyl sulfate (SDS), sodium deoxycholate (SDC) and Triton X-100 combined with heating. This was followed by in-gel digestion and tryptic cleavage for proteomic analysis. This method resulted in high proteomic reproducibility, enabling the comparison of inter-regional brain cortex tissue by bioinformatic analyses. Ingenuity Pathway Analysis and PANTHERdb were utilized to reveal differential regulation of signaling pathways between brain regions (Figure 1). These data provide a rich source of information for researchers and clinicians investigating specific areas of the human brain. Notably, there were similarly activated or inhibited signaling pathways in different brain regions. This indicates commonalities in the underlying molecular signaling pathways in various functional areas of the brain. The mapping of these brain region proteomes may help elucidate neurological processes and identify potential targets for therapeutics.

## 2. Results

### 2.1. Optimized Protocol to Evaluate the Proteome of Formalin-Fixed Human Brain Tissue

**Figure 2:**
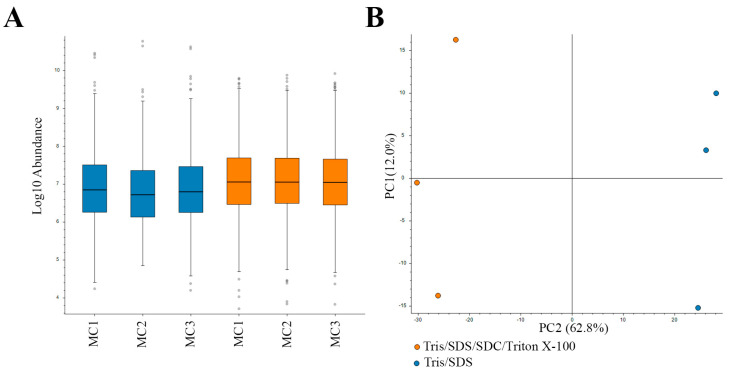
Label-free quantitative (LFQ) proteomics of biological replicates (1–3) of motor cortex (MC) tissue subjected to two protein extraction methods. (**A**) Relatively equal loading of protein quantity (abundance) for LFQ proteomics. (**B**) Two-dimensional principal component analysis (PCA) reflects the data quality and similarities of the proteome profiles between each biological replicate subjected to two different protein extraction methods. Blue, Tris/SDS lysis buffer; orange, Tris/SDS/SDC/Triton X-100 lysis buffer.

In this study, we used a high Tris concentration extraction buffer, together with detergents and heating, to efficiently extract proteins and remove formalin crosslinks. For this protein extraction method, two buffers were compared: (i) Tris/SDS/SDC/Triton X-100 buffer and (ii) Tris/SDS buffer. The protein extraction methods were each applied to three biological replicate motor cortex samples (from separate donors). Following extraction, equal amounts of protein were prepared for in-gel digestion for mass spectrometry (MS) analysis as previously described [18]. Similar distributions of protein sample abundances were observed in the MS analysis (Figure 2A), and an FDR of 0.05 was set for confidence in the assessment of the dataset. Two-dimensional PCA analysis showed a high level of consistency amongst the samples analyzed. Biological replicates were grouped together by protein-extraction buffer (Figure 2B).

**Figure 3:**
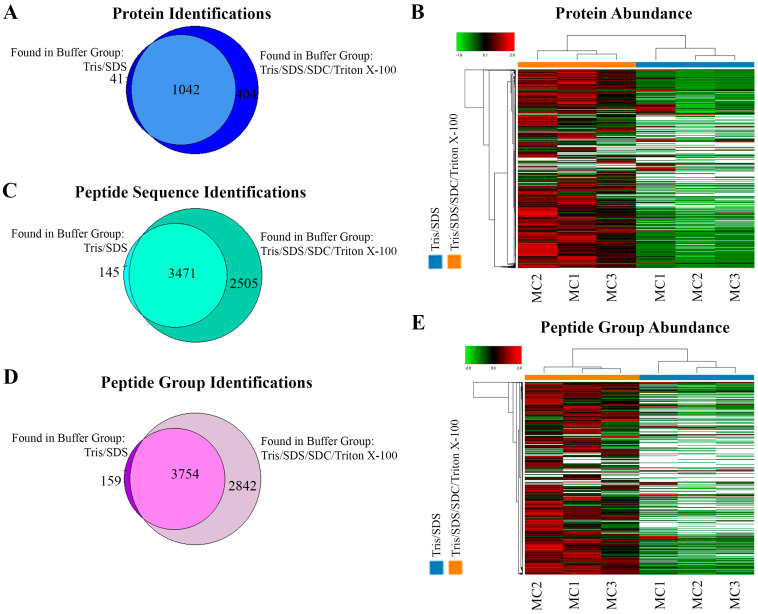
Tris/SDS/SDC/Triton X-100 protein-extraction buffer method resulted in more protein and peptide identifications and more abundant protein and peptide groups found by LFQ proteomics, in formalin-fixed human motor cortex tissue. Proteomic analyses of the two different lysis buffers were compared (Tris/SDS and Tris/SDS/SDC/Triton X-100). Venn diagram of motor cortex tissue identifying unique and common (**A**) proteins, and (**B**) heat map of proteomic clustering showing higher protein abundance patterns with the Tris/SDS/SDC/Triton X-100 lysis buffer. Venn diagrams of motor cortex tissue identifying unique and common (**C**) peptide sequences and (**D**) peptide groups. (**E**) Heat map of proteomic clustering showing higher peptide group abundance patterns with the Tris/SDS/SDC/Triton X-100 lysis buffer. Three biological replicates of formalin—fixed human motor cortex were assessed per protein extraction buffer analysis using Proteome Discoverer 2.4 software. MC1-3, motor cortex samples 1–3. Orange, Tris/SDS/SDC/Triton X-100 lysis buffer; blue, Tris/SDS.

Although a similar number of protein identifications and a large overlap were observed at the protein level between the two extraction methods (Tris/SDS/SDC/Triton X-100: 1446 vs. Tris/SDS: 1083) (Figure 3A), the Tris/SDS/SDC/Triton X-100 buffer showed distinctly more abundant protein patterns, which were sufficiently robust to cluster together like samples while distinguishing them from the Tris/SDS lysed samples (Figure 3B). This indicated that the catalogues of proteins extracted by each lysis buffer were similar among replicates, but distinguishable between lysis buffers, and that there was higher abundance of the commonly identified proteins in the Tris/SDS/SDC/Triton X-100 lysis buffer group. Despite the similar identification of number of proteins, the Tris/SDS/SDC/Triton X-100 extraction buffer resulted in a 65% higher number of identified peptide sequences compared to the Tris/SDS extraction buffer (Tris/SDS/SDC/Triton X-100: 5976 vs. Tris/SDS: 3616) (Figure 3C) and a 69% higher number of identified peptide groups compared to the Tris/SDS extraction buffer (Tris/SDS/SDC/Triton X-100: 6596 vs. Tris/SDS: 3913) (Figure 3D). Similarly to protein abundance patterns, peptide groups were also distinctly more abundant and robustly clustered together in the Tris/SDS/SDC/Triton X-100 buffer compared to the Tris/SDS buffer (Figure 3E). 

**Figure 4:**
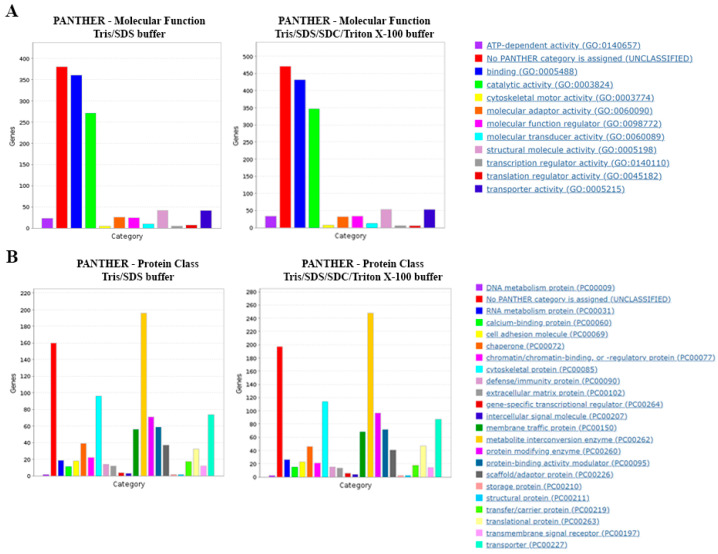
Gene Ontology (GO) analysis of proteins extracted by different lysis buffers. PANTHERdb was used to perform GO analysis on proteins extracted in lysis buffers Tris/SDS and Tris/SDS/SDC/Triton X-100. The lysis buffers extracted proteins with similar (**A**) molecular functions and (**B**) protein classes. Three biological replicates of formalin-fixed motor cortex tissue were analyzed for each lysis buffer tested.

To further interrogate the obtained proteomes with the two protein extraction methods, the datasets were subjected to Gene Ontology (GO) annotation using PANTHERdb. We observed similar distributions of protein classes and molecular functions for the two extraction methods (Figure 4A,B).

Taken together, these results indicate that both extraction buffers perform sufficiently well in extracting proteins from formalin-fixed human brain tissue for MS analysis. Although both buffers extracted similar numbers of proteins, there were greater protein abundance, greater peptide group abundance and higher percentages of identified peptide sequences and peptide groups using the Tris/SDS/SDC/Triton X-100 lysis method compared to the Tris/SDS lysis method. Therefore, the Tris/SDS/SDC/Triton X-100 lysis buffer method was selected for further experiments. 

#### Quantitative Reproducibility of Formalin-Fixed Human Brain Tissue

**Figure 5:**
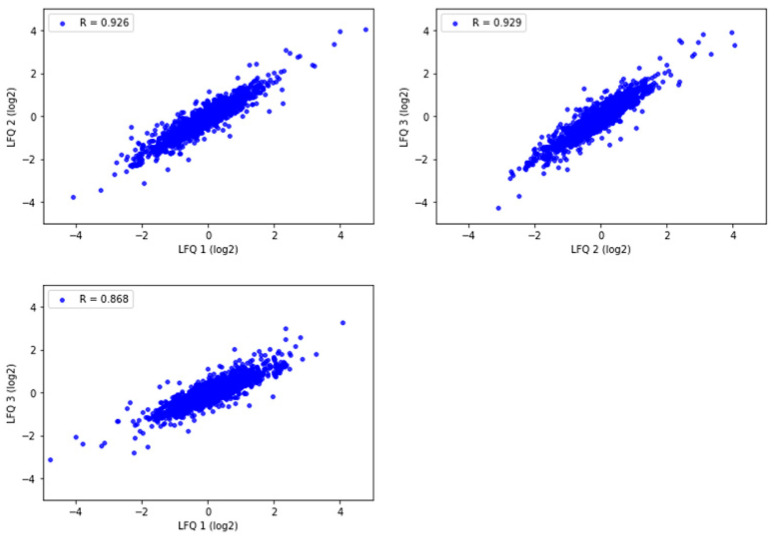
Pearson correlation of label free quantitation (LFQ) abundances between the three technical replicates of one representative motor cortex brain tissue sample analyzed by LC-MS/MS and Proteome Discoverer 2.4. R = 0.868–0.929. Pearson correlation coefficients were calculated and graphed using Python.

To assess the quantitative reproducibility among experimental replicates with the selected method, three technical replicates were prepared from one motor cortex tissue sample in Tris/SDS/SDC/Triton X-100 protein-extraction buffer. The Pearson correlation of protein abundance was evaluated for the commonly identified proteins. All replicates had a high level of correlation (R = 0.868–0.929) (Figure 5). This demonstrates high experimental reproducibility with the Tris/SDS/SDC/Triton X-100 lysis buffer. 

There were similar numbers of identified proteins and peptides groups in the technical replicates (Figure S1). Between 65 and 81% of proteins and 68 and 81% peptide groups were consistently identified in at least two of the replicates. An average of 2450 ± 338 proteins and 9277 ± 882 peptide groups were identified from 20 µg of tissue lysate per replicate. These results indicate that a robust number of proteins can be extracted from formalin-fixed brain tissue.

### 2.2. Inter-Regional Analysis of the Human Brain Cortex

**Figure 6:**
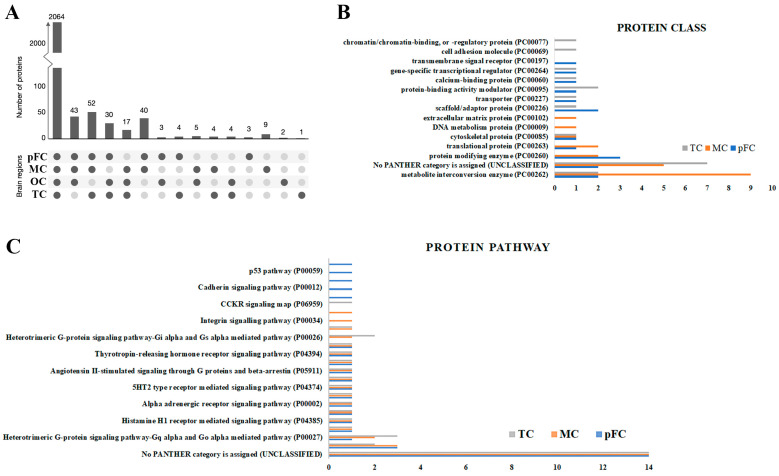
The majority of human brain proteins identified had low regional specificity and commonalities in enriched protein classes. (**A**) Histogram of the number of proteins identified in at least one region of the human brain tissue samples. Gene Ontology analysis of (**B**) protein classes and (**C**) protein pathways for the 20 most significantly enriched proteins using PANTHERdb (relative to the occipital cortex). pFC, pre-frontal cortex; MC, motor cortex; OC, occipital cortex; TC, temporal cortex.

The developed protocol using Tris/SDS/SDC/Triton X-100 protein-extraction buffer and heating together with in-gel tryptic digestion and MS analysis was applied to the analysis of 12 human brain tissue samples from four distinct neuroanatomical brain regions (pre-frontal cortex, motor cortex, temporal cortex, and occipital cortex). Label-free protein quantifications were obtained from each of the four distinct brain regions of the three donors. Label-free quantification was used to maximize the number of protein identifications per sample [19]. The majority of proteins identified had low region specificity (Figure 6A). The frontal (including motor cortex) and temporal lobe cortex are implicated in neurodegenerative diseases, whereas the occipital lobe cortex undergoes minor pathological changes in later stages [20]. Consistent with gene-expression-level studies, we compared protein levels and signaling pathway analyses in the pre-frontal/occipital lobe, temporal/occipital lobe, and motor/occipital lobe in the brain tissue samples for relative LFQ analysis. In general, there were greater degrees of significantly enriched proteins (abundance ratio *p*-value < 0.05) in the pre-frontal and motor cortexes (69.7% and 65.9%, respectively) than in the temporal cortex (57.5%). 

#### Gene Ontology Analysis

Using region normalized protein expression data, we identified the 20 most significantly enriched proteins in each brain region compared to the occipital cortex (Table S1). ‘Metabolite interconversion enzymes’ and ‘Translational proteins’ were generally the most represented protein classes according to GO annotation by PANTHERdb (Figure 6B), excluding proteins that could not be classified into PANTHERdb protein classes. The ‘Ubiquitin proteasome pathway’ and ‘Heterotrimeric G-protein signaling pathway-Gq alpha and Go alpha mediated pathway’ were the second and third most enriched protein pathways among all three brain regions compared to the occipital cortex region, respectively (Figure 6C).

**Figure 7:**
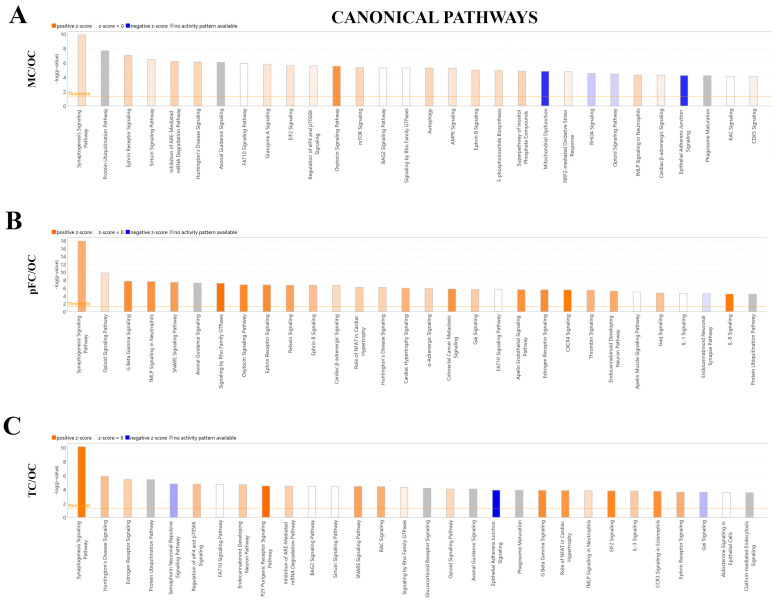
Significantly enriched canonical pathways identified QIAGEN Ingenuity Pathway Analysis (IPA). IPA top 30 canonical pathways in the (**A**) motor cortex vs. occipital cortex (MC/OC), (**B**) pre-frontal cortex vs. occipital cortex (pFC/OC), (**C**) temporal cortex vs. occipital cortex (TC/OC). Orange, positive z-score; white, z-score = 0; blue, negative z-score; gray, no z-score score predicted.

Ingenuity Pathway Analysis (QIAGEN) software was used to identify the significantly enriched canonical Pathways (*p*-value) and corresponding predicted activation states (z-score) of the total proteins identified (see Section 4.6). The top three most significant canonical pathways identified when comparing the motor cortex to the occipital cortex were ‘Synaptogenesis Signaling Pathway’ (*p*-value = 1.32 × 10^−10^, z-score: 0.784, 27 molecules), ‘Protein Ubiquitination Pathway’ (*p*-value = 2.28 × 10^−8^, z-score = N/A, 22 molecules), and ‘Ephrin Receptor Signaling’ (*p*-value = 9.11 × 10^−8^, z-score: 1.069, 18 molecules). ‘Synaptogenesis Signaling Pathway’ and ‘Ephrin Receptor Signaling’ were assigned positive z-scores, suggesting predicted activation of these canonical pathways when comparing the motor cortex to the occipital cortex (Figure 7A). 

The three most significant canonical pathways identified when comparing the pre-frontal cortex to the occipital cortex were ‘Synaptogenesis Signaling Pathway’ (*p*-value = 1.43 × 10^−18^, z-score: 1.826, 34 molecules), ‘Opioid Signaling Pathway’ (*p*-value = 1.43 × 10^−18^, z-score: 1.826, 23 molecules), and ‘G Beta Gamma Signaling’ (*p*-value = 2.00 × 10^−8^, z-score: 2.496, 17 molecules). All three pathways were assigned positive z-scores, suggesting activation of these canonical pathways when comparing the pre-frontal cortex to the occipital cortex (Figure 7B).

The three most significant canonical pathways identified when comparing the temporal cortex to the occipital cortex were the ‘Synaptogenesis Signaling Pathway’ (*p*-value = 7.49 × 10^−11^, z-score: 2.711, 24 molecules), ‘Huntington’s Disease Signaling (*p*-value = 1.34 × 10^−6^, z-score: 1.342, 17 molecules), and ‘Estrogen Receptor Signaling’ (*p*-value = 3.82 × 10^−6^, z-score: 1, 20 molecules). All three pathways were assigned positive z-scores suggesting activation of these canonical pathways when comparing the temporal cortex to the occipital cortex (Figure 7C).

IPA was also used to assign canonical pathways in the form of a bubble chart in order to identify pathway categories that were significantly similar or different between the brain regions and clusters of proteins associated with these categories. The most significant pathway categories identified in all three comparisons were ‘Neurotransmitter and Other Nervous System Signaling’, and ‘Organismal Growth and Development’. Notably, large clusters of proteins (27 for motor cortex vs. occipital cortex, *p*-value = 1.43 × 10^−10^; 34 molecules for pre-frontal cortex vs. occipital cortex, *p*-value = 1.43 × 10^−18^; 24 molecules for temporal vs. occipital cortex, *p*-value = 7.49 × 10^−11^) were involved in the ‘Synaptogenesis Signaling Pathway’. Compared to the occipital cortex, these pathways were consistently assigned a positive z-score (motor vs. occipital cortex, z-score = 0.784; pre-frontal cortex vs. occipital cortex, z-score = 1.826, temporal vs. occipital cortex, z-score = 2.711), suggesting predicted activation of this pathways in the motor cortex, temporal cortex and pre-frontal cortex when compared to the occipital cortex (Figures S2–S4). 

**Figure 8:**
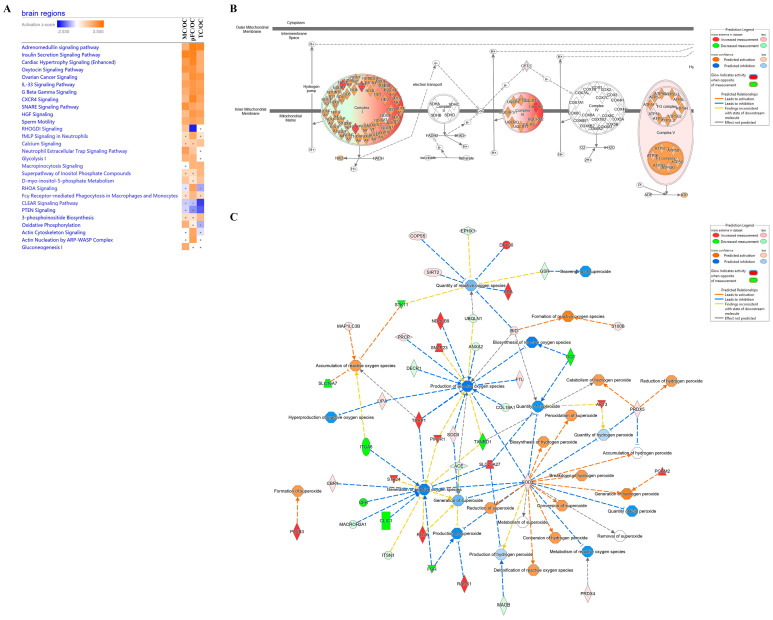
Comparative analysis of canonical pathways between brain regions. (**A**) Examples of similarly or differently activated canonical pathways between brain regions. (**B**) Oxidative phosphorylation pathway in the motor cortex compared to the occipital cortex. (**C**) Network of molecules differentially regulated in the motor cortex compared to the occipital cortex that are related to free radical species. Significant canonical pathways and molecules with absolute fold changes of at least 1.5 were included. Orange, positive z-score; white, z-score = 0; blue, negative z-score; gray, no z-score score predicted. Grey dot, not significant. Regions were compared to the occipital cortex. pFC, pre-frontal cortex; MC, motor cortex; OC, occipital cortex; TC, temporal cortex.

Comparative analysis of significant molecules that had at least an absolute 1.5 fold-change difference in activation values was carried out to reveal differential regulation of canonical pathways between brain regions compared to the occipital cortex (Figure 8A). Although several canonical pathways were similarly activated or inhibited among the brain regions compared to the occipital cortex, pathways with differential regulation between brain regions were further interrogated. ‘RHOGD1 signaling’ was predicted to be activated in the motor cortex (*p*-value = 3.97 × 10^−^^3^ and z-score = 1.6333), inhibited in the pre-frontal cortex (*p*-value = 2.29 × 10^−^^4^ and z-score = −2.449), and not significantly different in the temporal cortex. Energy production pathways were also differentially regulated. ‘Glycolysis I’ was predicted to be activated in the motor cortex (*p*-value = 1.72 × 10^−^^3^, z-score = 2) and pre-frontal cortex (*p*-value = 8.32 × 10^−^^4^, z-score = 2), but not significantly different in the temporal cortex. ‘Gluconeogenesis I’ was predicted to be activated in the motor cortex (*p*-value = 1.72 × 10^−^^3^, z-score = 2) but not significantly different in the pre-frontal and temporal cortexes. ‘Oxidative phosphorylation’ was predicted to be activated in the motor cortex (*p*-value = 5.96 × 10^−^^3^, z-score = 1.89) and not significantly different in the pre-frontal and temporal cortexes. 

Upon further interrogation of the oxidative phosphorylation pathway in the motor cortex compared to occipital cortex, complexes I, III, and V of the inner mitochondrial membrane were predicted to be activated (Figure 8B). We then searched this dataset further for molecules related to free radicals, given their relation to mitochondrial oxidative phosphorylation. We found 44 differentially regulated molecules in the motor cortex related to the inhibition or activation of free radical species (Figure 8C). 

## 3. Discussion

This proof-of-concept study provides an optimized lysis buffer for protein extraction from formalin-fixed human brain tissue to investigate proteins of interest and inter-regional expression patterns by proteomic analysis. Notably, we demonstrate that this method is suitable to identify the underlying cellular signaling pathways by LFQ proteomics. We propose that 600 mM Tris/SDS/SDC/Triton X-100 protein-extraction buffer combined with heating at 90 °C, agitation, and in-gel tryptic digestion enables in-depth proteomics analysis of formalin-fixed human brain cortex tissue. This optimized method enabled us to use a low quantity of protein as starting material for MS analysis. Overall, we identified an average of 2450 ± 338 proteins and 9277 ± 882 peptide groups per sample and obtained reliable technical replication of these proteins and peptide groups. The ’Ubiquitin Proteasome Pathway’ and ‘Translational Proteins‘ and ‘Metabolite Interconversion Enzymes’ were the most clearly enriched protein pathway and classes of the 20 most enriched proteins identified in the pre-frontal, motor and temporal cortices compared to the occipital cortex according to gene ontology analysis. Further, synaptogenesis signaling was consistently predicted to be activated in each of the studied brain regions relative to the occipital cortex. Comparative analysis revealed differential proteomic regulation of energy metabolism pathways among different brain regions, and we further provide evidence of metabolic molecular regulation within the human brain cortex.

Previous studies have indicated that fresh frozen tissue is preferred to formalin-fixed tissue due to concerns about formaldehyde-induced crosslinking and degradation during processing. However, the availability of fresh frozen tissue is often limited. Formalin crosslinking also inactivates pathological and biochemical processes and may be required for the investigation of organ specimens affected by pathogens [21]. As such, formalin fixation may be an essential step. Studies have investigated the potential of formalin-fixed tissue for proteomic analyses, increasingly demonstrating comparability in proteomics datasets [22,23,24,25,26,27,28]. High quality of sample preparation and protein extraction is essential for reliable proteomic analysis [27]. Several studies have reported successful results using heat or a barocycler [29] to reverse formaldehyde crosslinking. A recommended minimum 300 mM Tris hydrochloride (TrisHCl) concentration was suggested to achieve optimized FFPE proteomics analysis in mouse tissue [21]. Kawashima et al. proposed that at least two mechanisms may be involved in the Tris-catalyzed enhancement of protein extraction from FFPE tissue. Firstly, the Tris molecule may act as a scavenger to remove released formaldehyde, and secondly, Tris may act as a transamination catalyst directly breaking down the crosslinks [21]. We found that heating the samples at 90 °C in 600 mM TrisHCl and SDS protein-extraction buffer with agitation effectively reversed protein crosslinks in the formalin-fixed human brain tissue investigated in this study. Additional detergents included in the lysis buffer, SDC and Triton X-100, assisted in extracting increased protein yields to achieve in-depth proteomics datasets in a quick and suitable manner.

A major limitation encountered in clinical proteomics has been obtaining sufficient protein quantity for in-depth proteomic analysis. LFQ proteomic analysis of 200 µg of powdered, frozen brain tissue has been reported to detect an average of 3612 proteins per sample [4]. In the current study, only 20 µg of extracted protein from the formalin-fixed brain tissue was analyzed per sample. The average number of regional proteins identified in this study was 2199 proteins per sample. Hence, the protein extraction method achieved relatively in-depth proteomic analysis for the quantity of protein analyzed by MS. Proteomic analysis of matched formalin-fixed, paraffin embedded (FFPE) and fresh frozen meningioma tissue also resulted in a similar number of proteins and similar quality of mass spectra, but there were differences in chemical and post-translational modifications, which was not the focus of this study [30]. Most proteins identified in the brain regions from this study had low regional specificity, which is consistent with gene expression in the human brain reported in the human protein atlas (v22.proteinatlas.org) [31]. This indicates that protein extraction using Tris/SDS/SDC/Triton X-100 lysis buffer with heating and in-gel tryptic digestion is a robust method for LFQ proteomic analysis of formalin-fixed human brain tissue. This also demonstrates that successful MS and bioinformatic analyses can be performed on protein extracted from formalin-fixed human brain tissue. With formalin-fixed human tissue becoming more readily available, this method has great potential for future global proteomic analyses. 

Importantly, this method demonstrated the feasibility of using formalin-fixed human brain tissue to identify underlying cellular pathways by LC-MS/MS and label-free proteomics. Previous studies focused on the integration of proteomics and omics data or neuroanatomical-specific proteins to investigate the human brain [3,4,6,7,32]. This research expands our understanding of the human brain by identifying inter-regional specific signaling pathways. 

Evaluating brain region specific changes, we found ‘Neurotransmitters’, ‘Other Nervous System Signaling’, and ‘Synaptogenesis’ were the most abundantly represented canonical pathways. This was expected for brain tissue and confirms the robustness of the protein extraction employed in this study. Moreover, the enriched signaling pathways that were predicted to be activated in our study are consistent with the current literature and offer several potential proteins of interest for future investigations. For example, in the motor cortex, differential sirtuin expression patterns have been identified in post-mortem tissue of patients with motor neuron degeneration from amyotrophic lateral sclerosis (ALS) [33]. The enriched sirtuin signaling pathway identified in this study can be further scrutinized to identify proteins of interest relative to the motor cortex. It was unsurprising to find that sirtuin signaling predominated the motor cortex, given its broad role in glucose and lipid metabolism, and that the motor cortex controls physical movement, whereas the occipital cortex is generally responsible for vision [34]. Furthermore, fine movement is triggered by the neurons in the motor cortex through the corticospinal tract, which requires ephrins and their Eph receptors for topographical mapping of the corticospinal tract [35]. Ephrins and Eph receptors function within the ephrin receptor signaling pathway, which in this study, was enriched and predicted to be activated in the motor cortex relative to the occipital cortex. Additionally, strategies to inhibit RHOA signaling improve axonal regeneration of injured motor axons [36] and delay onset and extend survival [37] in neurodegenerative disease models of ALS. This is consistent with our predicted inhibition of RHOA signaling in healthy brain tissue in the motor cortex relative to the occipital cortex. The occipital cortex may be a suitable comparative control tissue for future investigations of RHOA signaling. Thus, this dataset provides a rich resource with which to investigate signaling pathways specific to movement in the motor cortex. 

Our results corresponded well to positron emission tomography imaging regarding elevated aerobic glycolysis in the pre-frontal cortex of the normal human brain, which differs among brain regions [38]. In contrast, this study predicted that glycolysis signaling was most activated in the motor cortex, which may not be related to the level of energy metabolism in the brain [38]. General regional differences in glucose metabolism in the brain were consistent with the literature [39]. The main energy source for the brain is oxidative phosphorylation, which is also a source of free radicals [40]. An insufficient energy supply can increase mitochondrial free radical production [41]. The motor cortex had the highest predicted activation of oxidative phosphorylation and of energy production pathways (i.e., glycolysis and gluconeogenesis). It is possible that there is a cooperative balance between these energy pathways to compensate for the increased metabolic demand, as evidenced by the apparent balanced molecular regulation of free radical species. 

The pre-frontal lobes are involved in regulating affect, personality, mood, and social and moral reasoning, amongst others [42]. The broad functions of the pre-frontal lobes likely contribute to the lack of predicted inhibition of the top pathways in the pre-frontal cortex relative to the occipital cortex. Dysregulated motivation is related to pre-frontal cortical opioids [43]. Opioid signaling was the second most enriched canonical pathway in the pre-frontal cortex tissue in this study and was predicted to be activated in the pre-frontal and temporal cortexes but inhibited in the motor cortex (vs. occipital cortex). Interestingly, our findings were consistent with RNA sequencing of post-mortem, pre-frontal cortex tissue of opioid use disorder (OUD) patients, which found that transcripts were related to synaptic remodeling [44]. ‘Synaptogenesis’ and ‘Opioid Signaling’ were the most enriched canonical pathways in the pre-frontal cortex in this study, confirming the robustness of the proteomic approach and offering neuroanatomically or functionally related pathways that may be explored further. These findings also suggest that opioid regulation within the temporal lobe might be of interest. 

The molecular mechanisms of prostanoid signaling involve the heterotrimeric G-protein signaling pathways regulating the downstream Wnt signaling pathway [45,46]. These pathways were enriched in each of the regions analyzed compared to the occipital cortex, suggesting inter-regional commonalities in molecular functions. Targeting these pathways may ultimately affect function in several brain regions. Moreover, this molecular signaling affects neurite extension length and calcium levels in neuronal growth cones [47], both of which are important for neuronal function. Prostaglandin E2 G-protein-coupled receptor signaling is also involved in the dendritic cell’s life cycle [48]. Interestingly, the cell cycle pathway was also enriched in each of the brain regions studied compared to the occipital cortex. Similarly activated or inhibited pathways in this study could be investigated further to potentially identify master regulator proteins of dysregulated signaling pathways in disease-relevant brain regions. 

During the human brain’s development into and throughout adulthood, protein levels increase or decrease over time [4]. Improvements to this study could be made with more samples, or by comparing different ages or comparing by sex. Future dataset comparisons of the human brain’s proteome should account for sub-specific regional proteomes (i.e., the frontal cortex proteome for the medial frontal gyrus or dorsolateral pre-frontal cortex compared to the superior frontal gyrus, which have proteomic dysregulation in Alzheimer’s disease [8,49] and the alcoholic brain [50], respectively). Nonetheless, this study demonstrated that it is possible to perform a high-throughput proteomic study without the need for expensive mechanical equipment to maintain consistency in sample preparation and increased output. 

Despite the inherent issues of working with post-mortem human brain tissue, during which membrane breakdown and protein translocation may occur during the post-mortem-to-fixation interval [12], the simple and optimized protein extraction from formalin-fixed human brain tissue presented in this study provided high quality proteins for MS analysis. Our proteomics findings were consistent with other omics studies on fresh frozen human brain tissue, highlighting the robustness of this approach. The proteomic data generated in this study ultimately present a rich source of information for neuroscientists and clinical proteomics, and knowledge of the different cellular pathways that are being driven within the examined brain regions.

## 4. Materials and Methods

### 4.1. Tissue Collection 

As a proof-of-concept, three formalin-fixed, post-mortem brain donors were included for sample collection. Four distinct neuroanatomical regions were collected from each of the donor brains: the frontal pole cortex (pre-frontal cortex), motor cortex, temporal cortex, and occipital cortex. A total of 12 brain tissue samples were collected (n = 12). Brain tissue was obtained from the Macquarie Medical School at Macquarie University under the approval of the Macquarie University Human Research Ethics Committee (5201300835) in accordance with the Declaration of Helsinki and relevant local guidelines. No formal criterion of post-mortem interval time from death to formalin fixation was used. The donors had no known clinicopathological diagnosis. Each brain was dissected and collected by a neuroanatomist and neurosurgeon with about 20 years of experience in the field, and stored in 70% ethanol at 4 °C. Samples were obtained from four neuroanatomical regions of three donors. 

### 4.2. Protein Extraction from Formalin-Fixed Tissue

A 1:10 ratio of tissue (mg) per extraction buffer (µL) was used with a minimum of 10 mg of brain-tissue starting material per sample. Each piece of tissue was chopped with a scalpel into smaller pieces prior to Dounce homogenization in cold protein-extraction buffer. Buffer was supplemented with protease and phosphatase inhibitors to a final concentration of 1X. Homogenate was transferred to a 1.5 mL microcentrifuge tube and kept on ice. Intermittent vortexing was repeated for 10 min. Homogenates were passed 5 times through a 25-gauge needle, followed by 3 times through a 30-gauge needle. Samples were incubated at 90 °C for 120 min with agitation at 750 rpm to remove the crosslinking which occurs with formalin fixation. Then samples were bath sonicated for 20 min. Lysates were clarified by centrifugation for 20 min at 16,000× *g* at 4 °C. The supernatant containing extracted proteins was collected and further processed. Total protein concentration was determined using the bicinchonic acid (BCA) method with bovine serum albumin as a standard, and samples were frozen as aliquots at −80 °C until further processing. Aliquots of lysate were diluted to be compatible with the protein quantitation assay, and the dilution factor was accounted for in determining the final protein concentration. 

### 4.3. Protocol Optimization

High concentration tris (hydroxymethyl) aminomethane hydrochloride (TrisHCl; referred to as Tris in manuscript) and sodium dodecyl sulfate (SDS) protein-extraction buffers with or without sodium deoxycholate (SDC), Triton-X-100, sodium chloride (NaCl), and ethylenediaminetetraacetic acid (EDTA) were compared to identify the buffer that extracted the most proteins. Equal weights of motor cortex tissue from each of the donors (n = 6) were prepared in parallel, as previously described. Buffers contained (i) 600 mM TrisHCl, 2% SDS, 150 mM NaCl, 0.5% SDC, 1 mM EDTA, 1% Triton X-100, phosphatase and protease inhibitor, pH 8.0, (referred to as Tris/SDS/SDC/Triton X-100 buffer) or (ii) 600 mM TrisHCl pH 8.0, 2% SDS, supplemented with protease and phosphatase inhibitor (referred to as Tris/SDS buffer). Three biological replicates for each buffer were prepared for protein analysis by mass spectrometry. 

The buffer that extracted the most proteins, as determined by MS analysis, was selected for further analysis. For multiregional brain proteome analysis, tissue from the motor, pre-frontal, temporal, and occipital cortexes (each approximately 15 mg of tissue in 150 µL buffer) from each of the three donors were prepared, as previously described, totaling 12 samples. 

Following extraction, equal amounts of protein lysates (20 µg) were mixed with Laemmli sample buffer and DTT (1X) and incubated for 5 min at 95 °C. The protein was loaded into Mini Protean TGX (4–15%) gels (BioRad) and electrophoresed for approximately 10 min at 200 Volts. Gels were then stained with Coomassie Blue for protein visualization. 

### 4.4. Protein Digestion of Brain Tissues

Proteomic procedures were carried out as recently described [18] with slight modifications. LC-MS/MS analysis of extracted proteins was performed. Samples were separated by SDS-PAGE gel electrophoresis (BioRad) for in-gel reduction with 10 mM DTT, alkylation with 55 mM IAA, and trypsin digestion (1:50 enzyme:protein) overnight at 37 °C (V5111, Promega). Extracted peptides were lyophilized and then resuspended in 0.1% formic acid (FA) for desalting using C18 OMIX tips (Agilent). Samples were lyophilized again and resuspended in 0.1% FA, bath-sonicated for 20 min, and then centrifuged at 14,000× *g* for 15 min to remove insoluble debris, and analyzed by LC-MS/MS. 

### 4.5. LC-MS/MS Analysis and MS Data Processing

The Ultimate 3000 nanoLC (Thermo Fisher Scientific) fitted with the Acclaim PepMap RSLC column particle size of 2 μm, diameter of 0.075 mm, and length of 150 mm (Thermo Fisher Scientific) was used, employing a 120 min gradient (2–80% *v*/*v* ACN, 0.1% *v*/*v* FA) running at a flow rate of 300 nL/min to separate peptides. Subsequently, eluted peptides were ionized into the Q Exactive Plus mass spectrometer (Thermo Fisher Scientific) that had an electrospray source fitted with an emitter tip 10 μm in diameter (New Objective) and maintained at 1.6 kV electrospray voltage. The capillary temperature was set to 250 °C. A data-dependent “Top 10” method operating in FT acquisition mode with HCD fragmentation was used for MS/MS fragmentation to select precursor ions. On the Q Exactive Plus, FT-MS analysis was carried out at 70,000-times resolution with an AGC target of 1 × 10^6^ ions in full MS and a maximum injection time of 30 milliseconds. Additionally, MS/MS scans were carried out at 17,500-times resolution with an AGC target of 2 × 10^4^ ions with the maximum injection time set to 50 milliseconds. The ion selection threshold was set to 25,000 counts to trigger MS/MS fragmentation. HCD fragmentation was performed using an isolation width of 2.0 Da with a normalized collision energy of 27. 

Raw files were searched with Proteome Discoverer 2.4 (Thermo Fisher Scientific) against Uniprot FASTA database incorporating the Sequest search algorithm. Search parameters accounted for 20 ppm precursor ion tolerance and 0.1 Da MS/MS fragment ion tolerance. The search allowed for static modifications of cysteine carbamidomethylation and variable modifications of methionine oxidation, asparginine, and glutamine deamidation on acetylated N-terminal residues. Two missed cleavages were allowed for. The data were processed through Percolator for estimation of false discovery rates. Protein identifications were validated employing a q-value of 0.05. Protein identification required at least one unique peptide per protein. 

### 4.6. Statistical and Bioinformatic Analysis

Statistically significant differences in protein, peptide sequence, and peptide groups identified were calculated using Proteome Discoverer 2.4. Common proteins identified in the LFQ of technical replicates were evaluated for Pearson correlation; the coefficients were calculated using the Scipy (v1.9.1) library in Python based on the logarithm values of proteins’ abundance ratios with variance stabilization. The histogram and scatter plots were generated using the Matplotlib (v3.6.0) library in Python. 

The frontal (Iding motor cortex) and temporal lobe cortexes are implicated in neurodegenerative diseases, whereas the occipital lobe cortex undergoes minor pathological changes in later stages [20]. Consistently with gene expression level studies, we compared protein levels and signaling pathway analyses in the pre-frontal/occipital lobe, temporal/occipital lobe, and motor/occipital lobe in the brain tissue samples for relative label-free quantification (LFQ). High-confidence proteins with abundance ratios having *p* < 0.05 were selected and included for pathway analysis. An absolute expression fold-change cutoff of 1.5 was used to identify analysis-ready molecules in Ingenuity Pathway Analysis (IPA). Data were analyzed with the use of QIAGEN IPA (QIAGEN Inc., https://digitalinsights.qiagen.com/IPA, accessed September–December 2022) [51]. Gene Ontology (GO) analysis of the proteomic results was conducted using online open-source software PANTHERdb (Protein ANalysis THrough Evolutionary Relationships) classification system 17.0, which annotated proteins to biological processes [52].

## Data Availability

The mass spectrometry proteomics data have been deposited on the ProteomeXchange Consortium via the PRIDE [53] partner repository with the dataset identifiers PXD039808 and 10.6019/PXD039808.

## Supplementary Materials

The following supporting information can be downloaded at: https://www.mdpi.com/article/10.3390/ijms24054283/s1.
