# Supplementary material for: Inter-Regional Proteomic Profiling of the Human Brain Using an Optimized Protein Extraction Method from Formalin-Fixed Tissue to Identify Signaling Pathways
Source: Int J Mol Sci. 2023 Feb 21;24(5):4283. doi: 10.3390/ijms24054283 (PMC10001664; doi:10.3390/ijms24054283)
Supplement: Supplementary file 1 [file ijms-24-04283-s001.zip › ijms-2217742-supplementary.pdf]

Supplementary Materials

Table S1. TOP 20 most enriched proteins (vs occipital cortex) Gene Symbol

Pre-frontal	Motor	Temporal
PLCB3	MRPL12	RGS20
PSMD1	AMY1A	DSG1
PSMD11	AMY1B	H1-1
PSMC5	AMY1C	RAB2B
MRPS18B	CARNS1	PTPRA
RPS15	DOHH	DOHH
MPP1	DYNLRB1	POF1B
RPL3	GCLC	RGS6
RPL38	GSTK1	MVB12B
ACTR10	COL2A1	PLCB3
ACTR2	POF1B	PSMD1
ACTR3B	RGS6	PSMD11
ADGRB2	IDI1	ACTR10
ARL6IP1	MDP1	ARL6IP1
AIF1L	MVB12B	AIF1L
FTO	PLCB3	AIMP2
AIMP2	PSMD1	AP3D1
AP3D1	PSMD11	BZW1
ATL1	PSMC5	BAD
BZW1	RPS15	C2CD4C

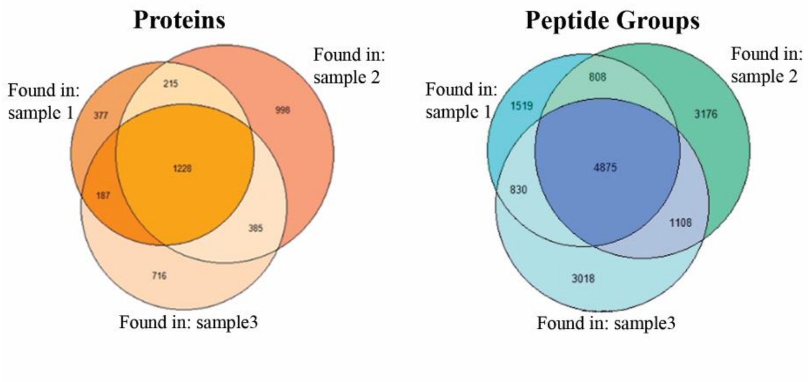

Figure S1. *Venn diagram of proteins and peptide groups identified in technical replicates prepared from one biological motor cortex brain tissue sample analyzed by LC-MS/MS and Proteome Discoverer 2.4.*

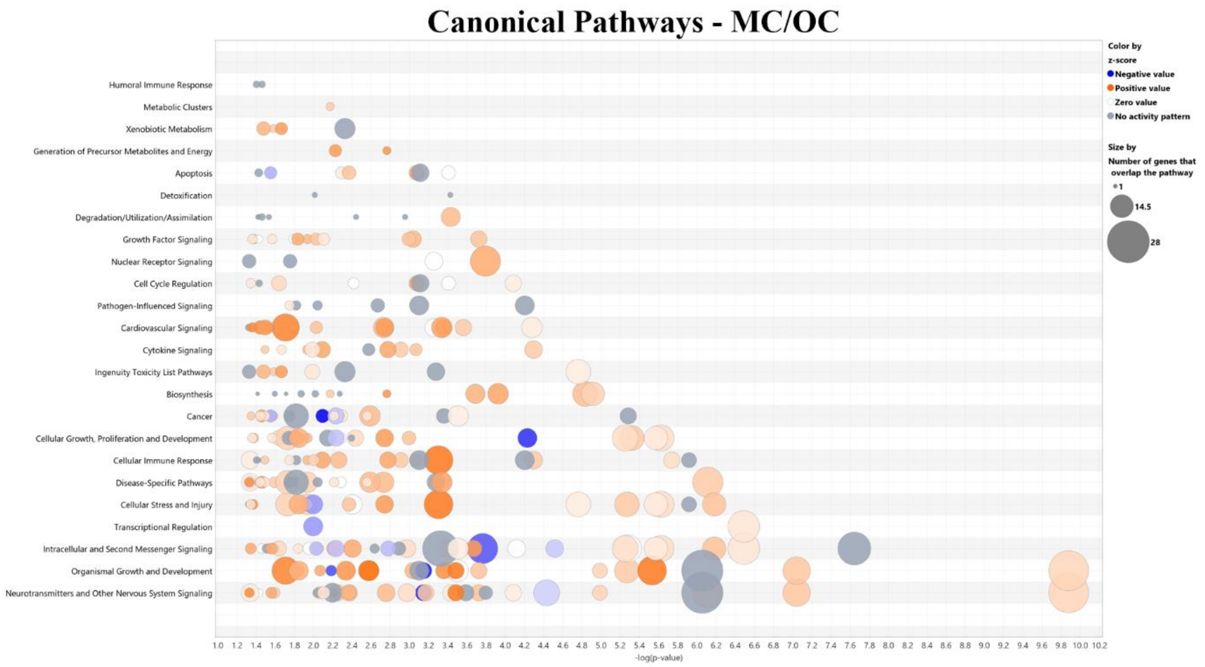

Figure S2. *IPA Bubble Chart of motor cortex compared to occipital cortex Canonical Pathways. Orange, positive z-score; Blue, negative z-score; White, z-score=0; Gray, z-score not predicted.*

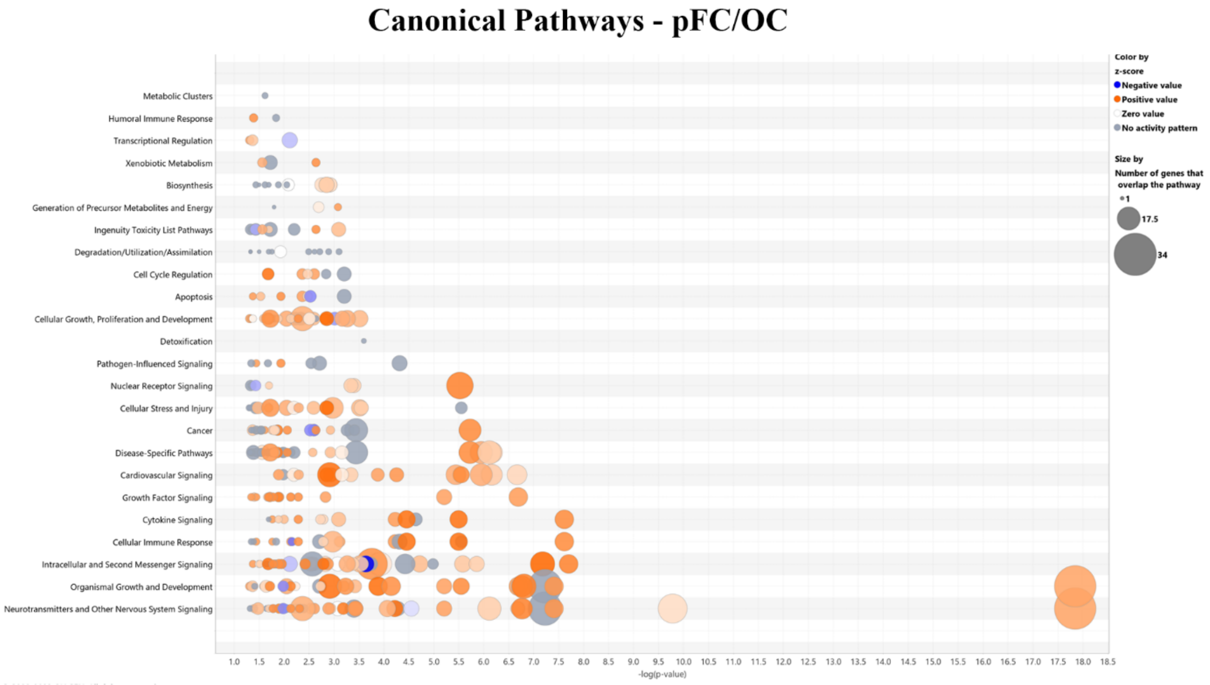

Figure S3 *IPA Bubble Chart of pre-frontal cortex compared to occipital cortex Canonical Pathways. Orange, positive z-score; Blue, negative z-score; White, z-score=0; Gray, z-score not predicted.*

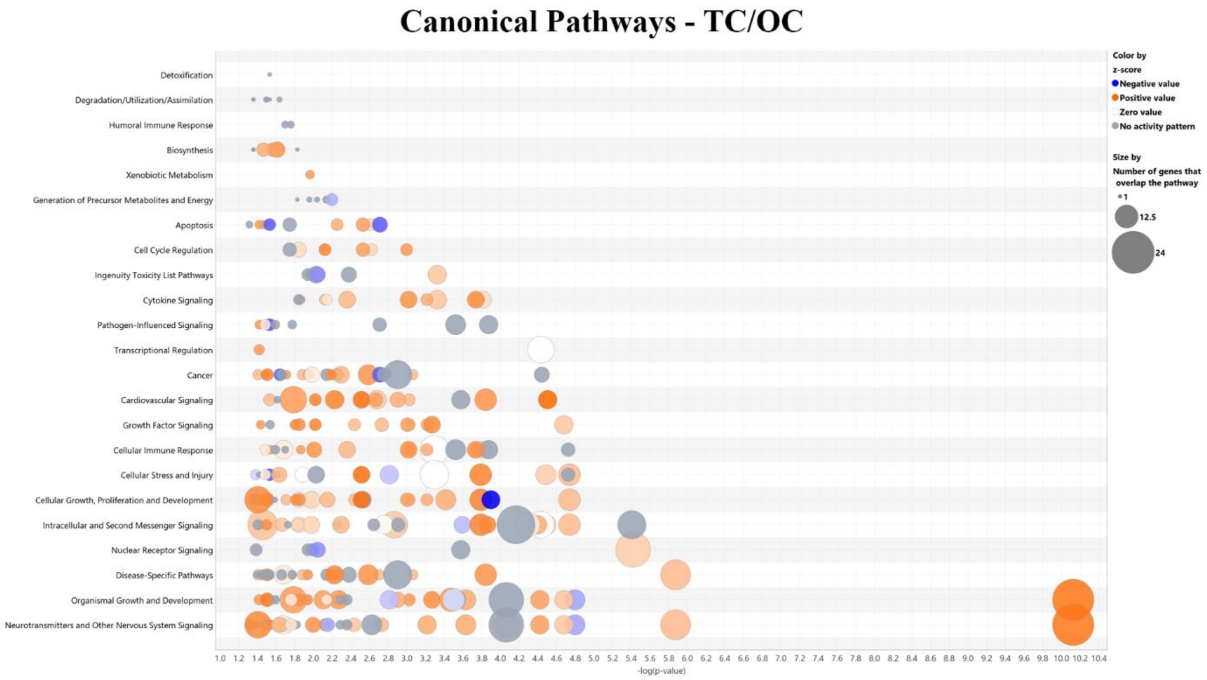

Figure S4 *IPA Bubble Chart of temporal cortex compared to occipital cortex Canonical Pathways. Orange, positive z-score; Blue, negative z-score; White, z-score=0; Gray, z-score not predicted.*
